# Antibiotic resistance and *mec*A characterization of *Staphylococcus hominis* from filarial lymphedema patients in the Ahanta West District, Ghana: A cross‐sectional study

**DOI:** 10.1002/hsr2.1104

**Published:** 2023-02-07

**Authors:** Priscilla Kini, Solomon Wireko, Priscilla Osei‐Poku, Samuel O. Asiedu, Emmanuel K. A. Amewu, Ebenezer Asiedu, Ernest Amanor, Caleb Mensah, Mary B. Wilson, Amma Larbi, Kennedy G. Boahen, Augustina A. Sylverken, Katherine R. Amato, Alexander Kwarteng

**Affiliations:** ^1^ Department of Biochemistry and Biotechnology, College of Science Kwame Nkrumah University of Science and Technology Kumasi Ghana; ^2^ Kumasi Centre for Collaborative Research in Tropical Medicine Kwame Nkrumah University of Science and Technology Kumasi Ghana; ^3^ Department of Laboratory Technology Kumasi Technical University Kumasi Ghana; ^4^ Department of Theoretical and Applied Biology, College of Science Kwame Nkrumah University of Science and Technology Kumasi Ghana; ^5^ Department of Biomedical Engineering Koforidua Technical University Koforidua Ghana; ^6^ Department of Clinical Microbiology, School of Medical Sciences Kwame Nkrumah University of Science and Technology Kumasi Ghana; ^7^ Department of Anthropology Northwestern University Evanston Illinois USA

**Keywords:** antibiotics, filarial lymphedema, Ghana, *mec*A gene, resistance, *Staphylococcus hominis*

## Abstract

**Background and Aim:**

Filarial infections affect over 150 million people in the tropics. One of the major forms of filarial pathologies is lymphedema; a condition where the immune response is significantly altered, resulting in changes in the normal flora. *Staphylococcus hominis*, a human skin commensal, can also be pathogenic in immunocompromised individuals. Therefore, there is the possibility that *S. hominis* could assume a different behavior in filarial lymphedema patients. To this end, we investigated the levels of antibiotic resistance and extent of *mec*A gene carriage in *S. hominis* among individuals presenting with filarial lymphedema in rural Ghana.

**Method:**

We recruited 160 individuals with stages I–VII lymphedema, in a cross‐sectional study in the Ahanta West District of the Western Region of Ghana. Swabs from lymphedematous limb ulcers, pus, and cutaneous surfaces were cultured using standard culture‐based techniques. The culture isolates were subjected to Matrix‐Assisted Laser Desorption/Ionization Time of Flight (MALDI‐TOF) mass spectrometry for bacterial identification. Antimicrobial susceptibility testing (AST) was performed using the Kirby–Bauer method. *mec*A genes were targeted by polymerase chain reaction for strains that were cefoxitin resistant.

**Results:**

In all, 112 *S*. *hominis* were isolated. The AST results showed resistance to chloramphenicol (87.5%), tetracycline (83.3%), penicillin (79.2%), and trimethoprim/sulphamethoxazole (45.8%). Of the 112 strains of *S. hominis*, 51 (45.5%) were resistant to cefoxitin, and 37 (72.5%) of the cefoxitin‐resistant *S. hominis* haboured the *mec*A gene.

**Conclusion:**

This study indicates a heightened level of methicillin‐resistant *S. hominis* isolated among filarial lymphedema patients. As a result, opportunistic infections of *S. hominis* among the already burdened filarial lymphedema patients in rural Ghana may have reduced treatment success with antibiotics.

## 1 BACKGROUND

Lymphatic filariasis (LF) caused by lymph‐dwelling nematodes (*Wuchereria brancrofti*, *Brugia malayi*, and *Brugia timori)* remains a disease of poverty significantly in Africa, Asia, and some parts of South America.^1^ The infection has affected over 120 million people living in 72 countries in the tropics with countries in Africa contributing to the largest burden.^2^, ^3^ LF‐infected individuals are generally asymptomatic, but most affected patients experience some form of clinical pathologies, including lymphedema and hydrocele, leading to significant disability‐adjusted life years (DALYs).^4^ According to McPherson et al.,^5^ over 40 million people suffer from lymphedema and hydrocele with 17 million suffering from chronic lymphedema.^6^ Currently, the intervention recommended by the WHO through the GPELF rely on the large‐scale administering of mainly microfilaricidal drugs (MDA) to the endemic populations.^7^, ^8^ These microfilaricidal drugs such as ivermectin can lower MF loads in infected humans but do not typically target secondary bacterial and fungal pathogens. Bacteria and fungi are believed to be important opportunistic pathogens in patients with filarial lymphedema due to the presence of lesions on their limbs which serve as entry portals.^9^ Bacterial products of *Staphylococci* are known to be responsible for recurrent skin inflammation such as cellulitis.^5^ Moreover, previous studies^5^, ^9^, ^10^ have also indicted bacterial products (superantigens) in worsening filarial infections, as a result of high cytokine release and mast cell degranulation. These superantigens are highly indicative of *Staphylococci aureus* co‐infection and not necessarily of the coagulase‐negative bacteria. However, some virulent factors such as biofilm formation, that complicate infected wound management have been identified with the coagulase‐negative bacteria.^11^, ^12^

*Staphylococcus hominis* is one of the major *Staphylococcus* species found on the human skin and is mainly found in the axillae, perineal and inguinal areas.^13^ *S. hominis* is ranked third among the coagulase‐negative bacteria that are of clinical importance.^14^ A previous study by Kloos et al.^13^ implicated *S. hominis* in the skin and soft tissue infections in immunocompromised individuals. Methicillin‐resistant *S. hominis* (MRSHo) are all capable of causing infections and usually are more likely to show multiple resistance to antimicrobial agents than other coagulase‐negative *Staphylococci*.^15^ In addition to its occasional pathogenicity, *S. hominis* may be a reservoir of specific components of the methicillin resistance genetic element, *staphylococcal* cassette chromosome (SCCmec) that may be transferrable to more pathogenic staphylococcal species.^15^ The pathogenicity of *S. hominis* in immunocompromised individuals have been of grave concern recently.

In LF, the pathology of the disease leaves most patients immunocompromised,^5^ and this is believed to result in a change in the normal flora, enabling hitherto non‐pathogenic organisms to be pathogenic. A resistant opportunistic pathogen could complicate secondary bacterial infections among individuals living with filarial lymphedema. Given the pathogenicity of *S. hominis* in immunocompromised individuals and its niche, it is likely that LF individuals may provide fertile grounds for pathogenetic activities of *S. hominis* contributing to the aggravation of filarial lymphedema. Nevertheless, there is no empirical evidence to fully implicate this opportunistic bacterium in lymphedema progression and provide tailored management.

As an important step toward addressing this gap, this study sought to evaluate the levels of antibiotic resistance and extent of *mec*A carriage in *S. hominis* among individuals presenting with filarial lymphedema in rural Ghana. The impact of bacterial infections remains to be documented among individuals with lymphedema in Ghana, where about 20% of persons develop some form of pathology in LF‐endemic communities. Therefore, the potential impact of this information and associated therapeutics on this population is great. We hypothesized that *S. hominis* do play a critical role in secondary bacterial infections among filarial lymphedema patients and complicate wound management strategies.

## 2 MATERIALS AND METHODS

This study employed a cross‐sectional design conducted from January 2019 to January 2020. Data collection included interviews using a structured questionnaire and swab sample collection from legs and leg wounds.

### 2.1 Study area and sampling

The study was conducted in eight (8) lymphatic filariasis endemic communities in the Ahanta West District, namely: Dixcove, Achowa, Busua, Butre, Asemkow, Ampatano, Princess Town, and Akatakyi. This District is about 260 km to the West of Accra, the capital of Ghana, and lies between latitude 4.8895̊ N and longitude 1.9603̊ W. The District consists of coastal towns of about 673 sq. km with a population of 106,215. Fishing and farming are the major occupations among the populace.^16^ All participants between the ages of 18–70 years with filarial lymphedema and who have lived in endemic filarial communities for more than 10 years were included in the study. Children and adults outside the stated age range were excluded. Individuals within the age range presenting with lymphedema of non‐filarial origin, existing autoimmune diseases, and debilitating comorbidities were also excluded.

### 2.2 Ethical considerations

Ethical clearance was obtained from the Committee on Human Research, Publication and Ethics, School of Medicine and Dentistry, KNUST, with approval number (CHRPE/AP/191/18).

### 2.3 Data and sample collection

A well‐structured questionnaire was used to gather information on the study participants. The questionnaire included questions on socio‐demographic characteristics, the number of years spent in the community, the location of the lymphedema, the stage of lymphedema, the duration of the infection, the presence of wounds, and signs of wound infection. Stages of lymphedema were determined using the WHO seven‐staged system (Dreyer et al., 2002).^17^ Copan eswabs (Copan Diagnostics Inc.) were used for sample collection and transport. In patients with wounds, the sizes were measured with a meter rule and recorded. Before each swab sample was taken, the collection site was cleaned with normal saline water, and excess saline was removed with sterile gauze. The site was then allowed to dry. The tip of a sterile swab was rotated over a 1 cm^2^ area for 5 s, while exerting sufficient pressure to extract fluid from wound tissue. The swab samples were transported frozen to the Kumasi Centre for Collaborative Research in Tropical Medicine, KNUST for laboratory analysis.

### 2.4 Culture and identification

To ensure the integrity of the samples, they were examined for leakages and correct labeling. Samples were immediately cultured upon arrival in the laboratory. The swabs were plated on Columbia Naladix Acid (CNA) agar and incubated at 35°C–37°C for 18–24 h. After 24 h, colonies on plates with mixed colonies were subcultured on CNA for pure isolates. The isolates were then stored in a microbank system at −80°C. MALDI‐TOF MS was used to identify bacteria.^18^

### 2.5 Antimicrobial susceptibility test

The disc diffusion method of antimicrobial susceptibility testing was used. The isolated *S. hominis* from the MALDI‐TOF analysis were subjected to antimicrobial susceptibility testing (AST), according to the European Committee on Antimicrobial Susceptibility Testing (EUCAST) guidelines.^19^ An inoculum suspension of *S. hominis* was adjusted to the 0.5 McFarland standard and streaked on Mueller Hinton agar plates. The antibiotics: ciprofloxacin, erythromycin, tetracycline, gentamicin, clindamycin, penicillin, cefoxitin, vancomycin, chloramphenicol, and trimethoprim‐sulphamethoxazole were placed on the streaked plates and incubated aerobically at 35°C for 16–18 h. After the specified incubation time, the various zones of inhibition for antibiotics were measured and recorded, and the results were interpreted, according to EUCAST recommendations. Cefoxitin disk diffusion test was used in the evaluation of Methicillin resistance of the isolates, and polymerase chain reaction (PCR) was used in detecting the presence of *mec*A gene. For the cefoxitin disk diffusion test, *S. hominis* isolates were considered resistant when the measurement of the zone of inhibition was ≥24 mm and susceptible if measurements were ≤25 mm.

### 2.6 DNA amplification and gel electrophoresis

DNA amplification was done to detect *mec*A and *mec*ALGA251. Table S1 shows the primer sequences and base pair sizes used. Multiplex PCR was carried out on each sample, as previously described by Stegger et al.,^20^ using Applied Biosystem thermal cycler, USA. After DNA amplification, the amplicons were separated by agarose gel electrophoresis as previously described^18^ and the DNA bands were visualized by illumination with UV light and images photographed.

### 2.7 Statistical analysis

Data analysis was done using International Business Machines version 26. All categorical variables were expressed as frequencies and percentages. Chi‐squared and Fischer's Exact test were performed to determine the association between the duration of the wounds and number of *S. hominis* isolates from the wounds. Statistical significance was considered at *p* ≤ 0.05.

## 3 RESULTS

### 3.1 Sociodemographic characteristics of study participants

In this study, 160 participants were recruited across the eight study communities, using the non‐probability convenience sampling approach. Most of the participants were in the age category 35–44 years (30.0%), 55–64 (24.4%), and 65 and above (23.8%). Here, 112 (70.0%) of the participants were females. Farming (31.3%) and fish mongering (20.6%) were the major occupations pursued by the participants, however, 30.0% were unemployed. Table 1 shows the sociodemographic data of the study participants.

**Table 1:** Characteristics of study participants.

Variable		*n* (%)
Community	Achowa	4 (2.5)
	Akatakyi	23 (14.4)
	Ampatano	21 (13.1)
	Asemkow	25 (15.6)
	Busua	21 (13.1)
	Butre	25 (15.6)
	Dixcove	21 (13.1)
	Princess Town	20 (12.5)
Sex	Female	112 (70.0)
	Male	48 (30.0)
Age groups	18–24	2 (1.2)
	25–34	6 (3.8)
	35–44	48 (30.0)
	45–54	27 (16.9)
	55–64	39 (24.4)
	65 and above	38 (23.8)
Occupation	Unemployed	48 (30.0)
	Farming	50 (31.3)
	Fishing	13 (8.1)
	Fish mongering	33 (20.6)
	Trader	8 (5.0)
	Hairdresser	2 (1.3)
	Bar tender	4 (2.5)
	Driver	2 (1.2)
Stage of lymphedema	1	13 (8.1)
	2	54 (33.8)
	3	46 (28.8)
	4	15 (9.4)
	5	17 (10.6)
	6	10 (6.3)
	7	5 (3.1)
Presence of wound	No	73 (45.6)
	Yes	87 (54.4)

### 3.2 Presence of wound, wound characteristics, and *S. hominis* isolated

**Figure 1:**
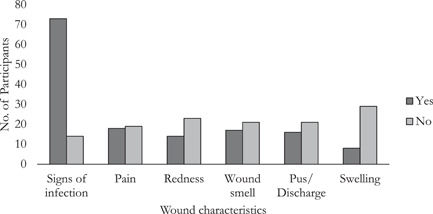
Presence of wound, wound characteristics, and presence of *Staphylococcus hominis*.

The majority of the study participants (33.8%) had Stage 2 lymphedema followed by Stage 3 (28.8%) with only 2.4% presenting with Stage 7. More than half of the participants (87, 54.4%) had wounds. A total of 56 (64.4%) of the participants with wounds were females, while 31 (35.6%) were males. The wounds were further categorized into two groups (i.e., with or without signs of infection); 73 (83.9%) of the participants with wounds showed some signs of infection (Figure 1). Regarding the wounds which showed signs of infection, 17 (23.3%) participants had a smelly wound, 14 (19.2%) participants had redness of wound, 16 (21.9%) participants had pus/discharge from their wounds, 8 (11.0%) had swollen wounds and 18 (24.7%) complained of painful wounds (Figure 1). A total of 112 *S. hominis* were isolated. 36 (32.1%) *S. hominis* were isolated from wound samples. To determine whether there was any association between the duration of a wound (determined from the responses to the questionnaire) and the presence of *S. hominis* (number isolated/wound), a Fisher's Exact test was performed. It was observed that there was a significant probability dependence (*p* = 0.004) between the duration of a wound and *S. hominis* (Table 2).

**Table 2:** Association of *Staphylococcus hominis* infection with duration of wound.

No. *S. hominis* per wound	Duration of wound (weeks)		*p*‐value
	4–8	>8	
2 microbes	3	6	
3 microbes	3	4	
≥4 microbes	0	10	0.004

### 3.3 AST results and *mecA*

**Figure 2:**
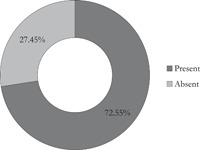
Presence of *mec*A gene in *Staphylococcus hominis* isolates.

**Figure 3:**
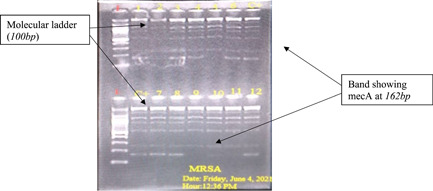
Gel electrophoresis showing *mecA* genes.

The majority of the *S. hominis isolates* were resistant to chloramphenicol (87.5%), tetracycline (83.0%), and penicillin (79.5%). They were more susceptible to gentamycin (83.0%), vancomycin (74.1%), clindamycin (70.5%), erythromycin (58.0%), and cefoxitin (54.5%) (Table 3a). Of the 112 *S. hominis* isolates, 51 (45.5%) were resistant to cefoxitin of which 37 (72.5%) of the cefoxitin‐resistant *S. hominis* haboured the *mec*A gene (Figures 2 and 3). Multi‐Drug Resistance (MDR) was found in the LF skin and wound isolates, which is defined as acquired non‐susceptibility to at least one agent in two or more antimicrobial categories Magiorakos et al.,^21^, ^22^ There was an association between antimicrobial resistance and carriage of staphylococcal *mecA* gene (Table 3b) (*p* = 0.0001).

**Table 3a:** Antimicrobial sensitivity testing for the isolated *Staphylococcus hominis*.

Antibiotic	S, *n* (%)	I, *n* (%)	R, *n* (%)
Cefoxitin	61 (54.5)	1 (0.9)	50 (44.6)
Chloramphenicol	9 (8.0)	5 (4.5)	98 (87.5)
Ciprofloxacin	93 (83.0)	0	19 (17.0)
Clindamycin	79 (70.5)	0	33 (29.5.)
Erythromycin	65 (58.0)	10 (9.0)	37 (33.0)
Gentamycin	93 (83.0)	0	19 (17)
Penicillin	23 (20.5)	0	89 (79.5)
Tetracycline	9 (8.0)	10 (9.0)	93 (83.0)
Trimethoprim‐sulphamethoxazole	42 (37.5)	19 (17)	51 (45.5)
Vancomycin	83 (74.1)	9 (8.0)	20 (17.9)

*Note*: I = intermediate to antibiotics tested; *n* = number of bacterial isolates; R = resistance to antibiotics; S = sensitive to antibiotics tested.

**Table 3b:** Comparison of antimicrobial drug resistance among *mecA* gene positive and negative isolates in LF.

Antibiotic	MRSA + isolates (*N* = 37)	Non‐MRSA isolates (*N* = 14)	All isolates (*N* = 51)
	% R	% R	*p*‐values
Cefoxitin	37 (72.5)	14 (27.5)	<0.0001
Chloramphenicol	37 (72.5)	14 (27.5)	<0.0001
Penicillin	37 (72.5)	14 (27.5)	<0.0001
Tetracycline	37 (72.5)	14 (27.5)	<0.0001

Abbreviations: LF, lymphatic filariasis; % R , percentage resistance; MRSA, methicillin‐resistant *Staphylococcus aureus*.

## 4 DISCUSSION

The potential threat of antibiotic resistance, particularly among commensals, continues to be a major concern for regions where filarial lymphedema is common.^18^ The prevalence of methicillin‐resistant *Staphylococcus aureus* (MRSA) among filarial lymphedema patients in Ghana has been documented.^18^ In that study, *Staphylococcus* species emerged as the most abundant among the Firmicutes, followed by *Streptococcal* species. Among the *Staphylococcus sp., S. hominis* was the most predominant bacteria isolated, followed by *S. epidermidis*. High isolates of *S. hominis* observed in the present study corroborates with the findings by Olszewski,^23^ who identified *S. hominis*, *S. epidermidis*, *S. capitis, S. xylosus*, *Micrococcus sp*., and *Bacillus cereus* as the most common isolates from tissue fluid, lymph and inguinal lymph nodes of people living with lymphedema. A possible explanation for this outcome is that *S. hominis* is one of the major *Staphylococcus* species found on the human skin, primarily in the axillae, legs, and arms,^23^ hence this may have influenced the microbial population found at the various locations of the body from which the swab samples were taken. In addition, *S. hominis* is the commonest and most diverse species isolated from an unpolluted marine environment.^13^

Filarial lymphedema development and progression are normally accompanied by recurrent episodes of acute dermatolymphagioadenitis, which may eventually result in wounds on the affected legs or hands.^5^, ^24^ In this study, the majority of participants were farmers and fishmongers hence, it is not uncommon that 87 out of 160 had wounds. Farmers and fishmongers with lymphedema usually do not use footwear and therefore, are predisposed to leg wounds. These wounds serve as entry points for bacteria and fungi that cause infection, thus complicating the morbidity. We observed that 73 (83.9%) of patients with wounds showed signs of infection, and the longer patients had wounds, the more likely they were to be infected. Previous field studies have reported that individuals presenting in the late stages of lymphedema are susceptible to frequent painful filarial attacks.^25^ However, the non‐existence of wound treatment protocol for filarial lymphedema patients could lead to patients taking medications, primarily antibiotics, in search of relief.

In Ghana, most bacterial isolates have acquired resistance due to the unregulated purchase of unprescribed antibiotics and/or inappropriate use of these antibiotics.^26^ From this study, the *S. hominis* isolates were resistant to chloramphenicol (87.5%), tetracycline (83.0%), penicillin (79.5%), and trimethoprim/sulphamethoxazole (45.5%). Resistance to these various antibiotics of different classes is indicative of multidrug resistance. The pattern of resistance among the patients to these antibiotics, especially resistance to tetracycline, is worrying given that tetracycline‐based antibiotics have extensively been used against lymphatic filarial infections in the study communities. Tetracycline‐based treatment such as doxycycline has been reported to be effective against the endosymbiont Wolbachia bacteria and led to improved leg condition among lymphedema patients.^27^ Therefore, there is a need to evaluate antimicrobial resistance patterns in these LF‐endemic communities, especially where these antibiotics have previously been explored as effective LF treatment options.

Of note, 51 of the isolates showed resistance to cefoxitin which could be indicative of the presence of MRSA. The additional resistance observed with chloramphenicol, tetracycline and trimethoprim/sulphamethoxazole re‐inforce the possible presence of MRSA. Thus, these antibiotics may not be effective in the empirical management of secondary infections among filarial lymphedema patients, especially with the non‐existent wound treatment protocol for filarial lymphedema patients. Furthermore, several isolates of *S. hominis* in certain conditions have been reported to possess vancomycin resistance gene Won and Kim.^28^ Hence, it is of relevance to highlight the sensitivity of the isolates to vancomycin. Based on this finding, vancomycin can be an effective agent in the management of LF‐related wound infections.

Recent reports revealed that *S. hominis* strains harbor many resistant genes and may have several mechanisms for its pathogenicity, including adhesion to epithelial cells and the invasion activity of extracellular toxins, causing damage to the host epithelium.^14^ In addition, other studies report the formation of biofilm as a characteristic virulent factor for coagulase‐negative bacteria such as *S. hominis and S. epidermidis*.^11^, ^12^ The primary mechanism of resistance reported in coagulase‐negative staphylococcus is the possession of the *mec*A gene.^29^ In this study, 72.5% of the 51 cefoxitin‐resistant isolates were positive for the *mec*A gene. This finding is consistent with another study in which all *S. hominis* subsp. *novobiosepticus* isolates had the *mec*A gene.^30^ Unfortunately, subtyping was not done in this study to establish if they are *novobiosepticus*.

The limitation of this study is that the antimicrobial resistance gene goes beyond the problem of a single resistant bacterium or an infected patient. Therefore, there is the possibility that several bacteria could have their genetic composition altered with the potential of community‐acquired antimicrobial resistance. Additionally, with the associated vancomycin resistance gene being reported in some *S. hominis* strains, it will be of great relevance to further investigate the vanA, vanB, and vanC genes in such strains. Next, the culture‐based approaches allowed for studying a minority of the microbiome population. Therefore, future studies may be warranted to use whole genome sequencing approaches to provide deep insight into the taxonomic composition and genetic capabilities of the microbiome associated with filarial lymphedema. This will bring us closer to fulfilling the second goal of the Global Program for Eliminating Lymphatic Filariasis, specifically focusing on morbidity management. Furthermore, there will be a need for subsequent studies pertaining to the treatment and follow‐ups of these patients to help validate our findings that show *S. hominis* as an opportunistic pathogen implicated in LF‐related wound infection.

## 5 CONCLUSION

S. *hominis* was common among filarial lymphedema patients in the Ahanta West District, Ghana. The antimicrobial susceptibility testing in this study revealed that most of the *S. hominis* isolates were positive for *mec*A and resistant to common antibiotics in Ghana. It is possible that *S. hominis*, which is ordinarily a commensal, acquired new resistance factors in filarial lymphedema patients, contributing to the challenges associated with filarial lymphedema management, particularly in those with chronic wounds. Thus, timely identification of such emerging pathogenic microbes and knowledge of their susceptibility to commonly used antibiotics in rural Ghana will offer significant milestones for filarial researchers, clinicians, and local health workers involved with managing secondary bacterial infections associated with filarial lymphedema. The findings from this study will offer critical insight into antimicrobial stewardship and management protocols for human filarial lymphedema, especially in resource‐limited settings.

## AUTHOR CONTRIBUTIONS

**Priscilla Kini**: Formal analysis; methodology; writing—review & editing. **Solomon Wireko**: Formal analysis; methodology; writing—original draft; writing—review & editing. **Priscilla Osei‐Poku**: Formal analysis; methodology; writing—original draft. **Samuel Opoku Asiedu**: Formal analysis; methodology; writing—review & editing. **Emmanuel Kobla Atsu Amewu**: Formal analysis; methodology; writing—review & editing. **Ebenezer Asiedu**: Formal analysis; methodology; writing—review & editing. **Ernest Amanor**: Formal analysis; methodology; writing—review & editing. **Caleb Mensah**: Formal analysis; methodology; writing—review & editing. **Mary Boapomah Wilson**: Formal analysis; methodology; writing—review & editing. **Amma Larbi**: Formal analysis; methodology; writing— review & editing. **Kennedy Gyau Boahen**: Data curation; formal analysis; investigation; methodology; supervision; writing—review & editing. **Augustina Angelina Sylverken**: Supervision; writing—review & editing. **Katherine Ryan Amato**: Supervision; writing—review & editing. **Alexander Kwarteng**: Conceptualization; funding acquisition; investigation; methodology; project administration; resources; supervision; writing—review & editing.

## CONFLICTS OF INTEREST STATEMENT

The authors declare no conflicts of interest.

## TRANSPARENCY STATEMENT

The lead author Alexander Kwarteng affirms that this manuscript is an honest, accurate, and transparent account of the study being reported; that no important aspects of the study have been omitted; and that any discrepancies from the study as planned (and, if relevant, registered) have been explained.

## Supporting information

Supplementary information.

## Data Availability

The data sets used and/or analyzed during the current study are available from the corresponding author on reasonable request.
