# Supplementary material for: Antibiotic resistance and mecA characterization of Staphylococcus hominis from filarial lymphedema patients in the Ahanta West District, Ghana: A cross‐sectional study
Source: Health Sci Rep. 2023 Feb 7;6(2):e1104. doi: 10.1002/hsr2.1104 (PMC9904197; doi:10.1002/hsr2.1104)
Supplement: Supplementary file 1 — Supplementary information. [file HSR2-6-e1104-s001.docx]

Table S1. Primer sequences used in DNA Amplification

| Genes | Primer sequence | Amplicon  size | Reference |  |
| --- | --- | --- | --- | --- |
| mecA p42821 | TCCAGATTACAACTTCACCAGG | 162 bp | Stegger et al., 2012 |  |
| mecA p72822 | CCACTTCATATCTTGTAACG |  |  |  |
|  |  |  |  |  |
| mecALGA251 MultiFP 2825  mecALGA251 MultiRP 2826 | GAAAAAAAGGCTTAGAACGCCTC  GAAGATCTTTTCCGTTTTCAGC | 138 bp | Stegger et  al., 2012 |  |
